# Insights into the mechanism of the effects of rhizosphere microorganisms on the quality of authentic *Angelica sinensis* under different soil microenvironments

**DOI:** 10.1186/s12870-021-03047-w

**Published:** 2021-06-22

**Authors:** Lei Zhu, Hui Yan, Gui-sheng Zhou, Chun-hao Jiang, Pei Liu, Guang Yu, Sheng Guo, Qi-Nan Wu, Jin-ao Duan

**Affiliations:** 1 National and Local Collaborative Engineering Center of Chinese Medicinal Resources Industrialization and Formulae Innovative Medicine, Jiangsu Collaborative Innovation Center of Chinese Medicinal Resources Industrialization, State Administration of Traditional Chinese Medicine Key Laboratory of Chinese Medicinal Resources Recycling Utilization, Nanjing University of Chinese Medicine, Nanjing, China; 2 State Key Laboratory of Crop Genetics and Germplasm Enhancement, Nanjing Agricultural University, Nanjing, China

**Keywords:** *Angelica sinensis*, Rhizosphere microorganism, Metabolomics, quality, Correlation

## Abstract

**Background:**

*Angelica sinensis* (Oliv.) Diels (*A. sinensis*) is a Chinese herb grown in different geographical locations. It contains numerous active components with therapeutic value. Rhizosphere microbiomes affect various aspects of plant performance, such as nutrient acquisition, growth and development and plant diseases resistance. So far, few studies have investigated how the microbiome effects level of active components of *A. sinensis.* This study investigated whether changes in rhizosphere microbial communities and metabolites of *A. sinensis* vary with the soil microenvironment. Soils from the two main *A. sinensis-*producing areas, Gansu and Yunnan Province, were used to conduct pot experiments. The soil samples were divided into two parts, one part was sterilized and the other was unsterilized planting with the seedling variety of Gansu danggui 90–01. All seedlings were allowed to grow for 180 days. At the end of the experiment, radix *A. sinensis* were collected and used to characterize growth targets and chemical compositions. Rhizosphere soils were subjected to microbial analyses.

**Results:**

Changes in metabolic profiles and rhizosphere microbial communities of *A. sinensis* grown under different soil microenvironments were similar. The GN (Gansu non-sterilized), YN (Yunnan non-sterilized), GS (Gansu sterilized), and YS (Yunnan sterilized) groups were significantly separated. Notably, antagonistic bacteria such as *Sphingomonas*, *Pseudomonas*, *Lysobacter*, *Pseudoxanthomonas*, etc. were significantly (*p* < 0.05) enriched in Gansu soil compared with Yunnan soil. Moreover, senkyunolide I and ligustilide dimers which were enriched in GS group were strongly positively correlated with *Pseudomonas parafulva*; organic acids (including chlorogenic acid, dicaffeoylquinic acid and 5-feruloylquinic acid) and their ester coniferyl ferulate which were enriched in YS Group were positively associated with *Gemmatimonadetes* bacterium WY71 and *Mucilaginibater* sp., respectively.

**Conclusions:**

The soil microenvironment influences growth and level/type of active components in *A. sinensis*. Further studies should explore the functional features of quality-related bacteria, identify the key response genes and clarify the interactions between genes and soil environments. This will reveal the mechanisms that determine the quality formation of genuine *A. sinensis*.

**Supplementary Information:**

The online version contains supplementary material available at 10.1186/s12870-021-03047-w.

## Background

Geo-authentic Chinese medicinal materials are traditional Chinese medicine (TCM) selected through long-term clinical practice. They exhibit satisfactory curative effect, stable quality, and high profile. In particular, the radix *A. sinensis* possess traditional efficacies; for instance, it nourishes the blood, promotes blood circulation, regulates menstruation, and relieves pain. It was first recommended as a therapeutic option for gynecological diseases [1] and is currently utilized for general protection of the heart, immune booster, anti-arrhythmia, and anti-atherosclerosis, etc. [2]. In China, *A. sinensis* is largely produced in Gansu, Yunnan, Qinghai, and other places. Min County of Gansu Province has long been acknowledged as its traditional geoherb region with a cool and humid climate, abundant rainfall, mostly fertile cinnamon and black soil, which are ideal for *A. sinensis* growth. Terrestrial plants and soil microorganisms mutually benefit through strong interactions. Plants provide habitats and reliable carbon and energy sources for soil microorganisms, which directly or indirectly contribute to the yield and quality of the host plants. Also, they boost the hosts' resistance by improving soil mineral nutrition, production of phytohormones, degradation of phytotoxic compounds, inhibition of soil-borne pathogens, etc. [3, 4]. Numerous reports indicate that plant species, growth, and development, host genotypes, geographical location, are the main drivers of rhizosphere microbial diversity [5–7]. Notably, geographical position and cultivation practice are the key factors that determine microbiome variation in field conditions; however, under controlled greenhouse conditions, the microbial composition varies with soil source and genotype [6]. Similar results were reported in a large-scale longitudinal field study that compared changes in microbial communities across 27 maize inbred lines planted in three fields [7]. Contrarily, plant genetic effects had a remarkable impact on the rhizosphere microbiome, surpassing geographic location, plant age, and climatic events.

Compelling evidence indicates that plant–microbe interactions are vital for healthy plant growth [8]. Currently, the key interest to explore genuine TCMs is mainly geared on soil properties and rhizosphere microorganisms. Important breakthroughs have been made in the growth and yield improvement of famous medicinal materials, including *Panax ginseng*, *Rehmannia glutinosa,* and *Panax notoginseng*, etc. Changes in rhizosphere microbial communities and accumulation of toxic substances are greatly associated with the replanting failure of TCMs. For instance, reports show that soil-borne pathogen, *Fusarium* flourishes as plants grow, whereas antagonistic bacterium *Pseudomonas* and *Bacillus* show opposite trend [9, 10]. Although *Ilyonectria* was generally described as the main causative agent for ginseng rusty root disease, a recent study revealed the potential association of the oxidation and deposition of rhizosphere Fe, Al, and Mn driven by nitrate-dependent Fe (II)-oxidizing bacteria with rusty root disease [11]. Moreover, the build-up of the *Fusarium* genus and accumulation of toxic diisobutyl phthalate (DiBP) is inversely associated with the population of *Pseudomonas*, *Bacillus*, and *Burkholderia*, etc., contributing to negative feedback between the soil and ginseng [9, 12]. Similarly, the accumulation of phenolic acids in the rhizosphere of *R. glutinosa* potentially restrain the growth of *Pseudomonas* spp., but exert positive effects on mycelial growth, sporulation, and toxin production of *Fusarium oxysporum* [13]. Further research shows that the imbalance of beneficial (*Pseudomonas* spp.) and harmful (*Enterobacter* spp.) QS (quorum sensing) bacteria mediated by QQ (quorum quenching) bacteria in the rhizosphere, accounting for a key factor of *R. glutinosa* replant diseases [14].

*A. sinensis* is a typical alpine plant with high requirements for temperature (20 ~ 24 °C), light (long daylight) and, high elevation zones (2000 ~ 2500 m). The production and quality of radix *A. sinensis* deteriorate following a decrease in altitude. Consequently, it becomes more vulnerable to early bolting, and this decreases the drug efficacy. The instability of the breeding base is the second largest reason affecting its quality. The traditional wasteland seedling raising manner that adopted all along was environmentally unsound. Due to the policy of Grain for Green and ecological protection, rotation of land seedling and cultivated wasteland seedling are regarded as the main choices. However, they are immature to guarantee the quality of *A. sinensis*. Reports on soil microbial communities of authentic *A. sinensis* are scanty due to the hurdles posed by the unique living environmental needs. Previously, we found that the number of dominant microbial species and the population diversities of bacteria, actinomyces, and fungi in the rhizosphere of Gansu province were more compared to other non-geoherb regions, including Yunnan and Sichuan province. *Proteobacteria* and *bacteroidetes* were the dominant bacterial phyla throughout the growing season of *A. sinensis*, whereas fungal dominant phyla varied with growth periods. Furthermore, the plant weight, root length, root diameter, soil pH, rainfall, and climate temperature were the main factors contributing to the variation in microbial community composition of *A. sinensis* [15]. The most recent study on plant–microbe interactions mainly focused on succession cropping obstacles of medicinal plants, responses of plant–microbe to environmental stress, changes in rhizosphere nutrition conditions, root system biomass, and growth but provided little information on the accumulation of active constituents under different soil microenvironments [16]. There is an urgent need to elucidate the mechanism of the effects of rhizosphere microorganisms on the quality improvement of authentic *A. sinensis*, to acquire *A. sinensis* materials that is stable quality.

Herein, we prepared a pot experiment with four treatments: (i) GN-non-sterilized soil sampled from Gansu province, (ii) GS-sterilized soil sampled from Gansu province (iii) YN-non-sterilized soil sampled from Yunnan province, (iv) YS-sterilized soil sampled from Yunnan province, planting with the seedling variety of Gansu danggui 90–01. The four controls were without seedling implants. Through a combination of metabolomics and 16 s rRNA pyrosequencing technology, we aimed to explore (i) the potential variation of rhizosphere microorganisms, and the growth and quality of *A. sinensis* under different soil microenvironments, and (ii) establish whether the growth and quality of *A. sinensis* are positively impacted by changes in rhizosphere microorganisms.

## Results

### The morphology and quality of *A. sinensis* under different soil microenvironments

**Fig. 1:**
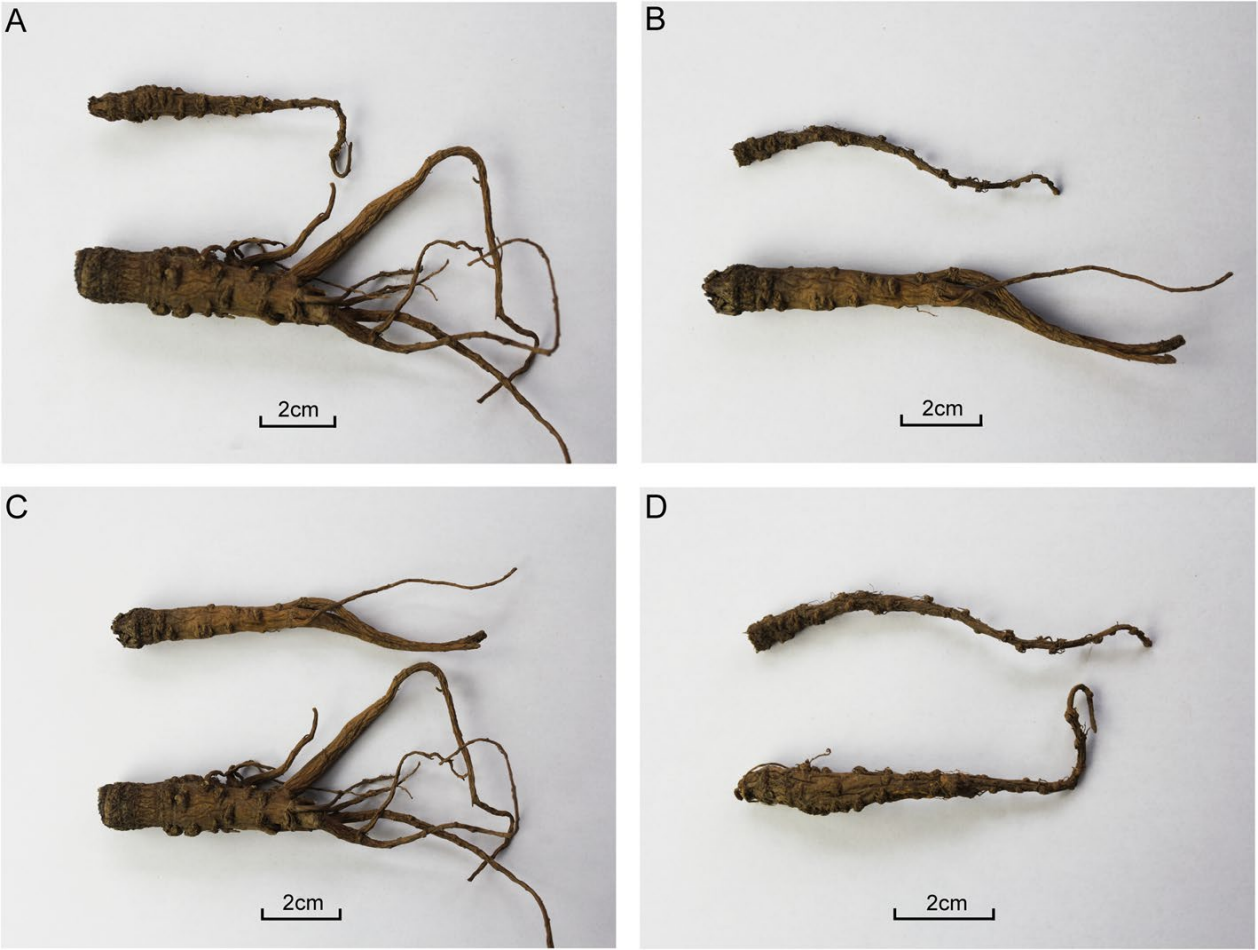
Photographs of *A. sinensis* grown in different soil microenvironments*.* **A**: GN (below) and YN (above) sample ; **B**: GS (below) and YS(above) sample; **C**: GN (below) and GS (above) sample; **D**: YN (below) and YS (above) sample. (Notice: GN represent Gansu non-sterilized group; YN represent Yunnan non-sterilized group; GS represent Gansu sterilized group; YS represent Yunnan sterilized group)

Regardless of whether the soil was sterilized or not, *A. sinensis* grown in Gansu soils (including GN and GS group) was significantly (*p* < 0.05) more branches and larger diameters than that grown in Yunnan soils (including YN and YS group) (Fig. 1A and B). Plants grown in sterilized soils displayed worse growth as indicated by the thinner rhizome, fewer number of branches and smaller diameter compared to those grown in non-sterilized soils (Fig. 1C and D). The survival rate of plants grown in non-sterilized soils (GN, 0.77 ± 0.09; YN, 0.63 ± 0.18; *n* = 6) was remarkably (*p* < 0.05) higher than that of plants grown in sterilized soils (GS, 0.48 ± 0.15; YS, 0.46 ± 0.19; *n* = 6). Further results showed that the average dry weight, root diameter, rootlet number, and root length of plants in GN group were significantly (*p* < 0.05) larger than that of plants of YN group. Plants of GS group showed substantially (*p* < 0.05) reduced average dry weight, root diameter and root length. The root diameter of plants in YN group was significantly (*p* < 0.05) higher than in YS group, and higher in GS group than in YS group (Table 1).

**Table 1:** Root system growth of *A. sinensis* from different soil microenvironments

Group	Average surviving rate /%	Average dry weight /g	Average root diameter /mm	Average rootlet number	Average root length /cm
GN	0.77 ± 0.09 a	2.94 ± 0.85 a	7.86 ± 1.66 a	6.94 ± 1.64 a	11.14 ± 2.34 a
YN	0.63 ± 0.18 a	0.88 ± 0.25 b	5.12 ± 0.88 b	1.72 ± 0.80 b	7.65 ± 0.70 bc
GS	0.48 ± 0.15 b	1.30 ± 0.62 b	6.14 ± 1.13 b	5.98 ± 2.01 ab	10.36 ± 1.13 c
YS	0.46 ± 0.19 b	0.36 ± 0.05 b	3.01 ± 0.74 c	1.00 ± 0.00 b	9.01 ± 0.79 b

Values are mean ± standard error (*n* = 6). Values with the same letter are not statistically signifificantly different, ANOVA, *p* < 0.05

To further explain the cause of differences in quality of *A. sinensis* among groups, medicinal components (phthalides and organic acids) and nutrients (amino acids, nucleosides and nucleobases) were compressively analyzed. The results showed that the contents of medicinal components were remarkably (*p* < 0.05) different among groups. Specifically, the level of chlorogenic acid and *Z*-ligustilide in *A. sinensis* grown on Yunnan soil was significantly (*p* < 0.05) higher than in plants grown on Gansu soil. The level of coniferyl ferulate and butylidenephthalide in the GN group was higher, whereas that of ferulic acid was lower than in other three groups. The level of senkyunolide A and butylphthalide in YN group was markedly (*p* < 0.05) higher than in the other groups. The level of senkyunolide I and senkyunolide H in YS group was significantly (*p* < 0.05) lower than in the other groups. However, the level of senkyunolide I and senkyunolide H was similar across GN, YN and GS groups (Table 2).

**Table 2:** Comparison of the analytes in different groups of *A. sinensis* samples (mg/g)

Group	Chlorogenic acid	Ferulic acid	Senkyunolide I	Senkyunolide H	Coniferyl ferulate	Senkyunolide A	Butylphthalide	Z-ligustilide	butylidenephthalide
GN	1.89 ± 0.15 c	0.48 ± 0.15 c	0.22 ± 0.09 a	0.08 ± 0.04 a	0.51 ± 0.42 a	0.02 ± 0.03 b	**-**	6.25 ± 1.34 b	0.35 ± 0.08 a
YN	3.62 ± 0.35 a	0.72 ± 0.11 b	0.18 ± 0.05 a	0.07 ± 0.02 a	0.08 ± 0.04 b	0.96 ± 0.33 a	0.35 ± 0.26 a	9.68 ± 1.49 a	0.23 ± 0.06 b
GS	2.05 ± 0.23 c	0.87 ± 0.07ab	0.23 ± 0.09 a	0.10 ± 0.04 a	0.04 ± 0.03 b	0.07 ± 0.09 b	**-**	6.96 ± 1.52 b	0.26 ± 0.09 b
YS	2.88 ± 0.43 b	0.97 ± 0.24 a	0.04 ± 0.00 b	0.01 ± 0.00 b	0.10 ± 0.07 b	0.32 ± 0.25 b	0.03 ± 0.04 b	11.01 ± 2.78a	0.07 ± 0.02 c

Values are mean ± standard error (*n* = 6). Values with the same letter are not statistically signifificantly different, ANOVA, *p* < 0.05

**-** Not detected

Previously, we found that *A. sinensis* possess high nutritive components including nucleosides, nucleobases and free amino acids [17]. The mean content of cytidine-5'-monophosphate accounted for 31.03% of the total nucleosides and nucleobases, whereas adenosine accounted for 27.68%, uridine for 17.84% and guanosine for 12.46%, respectively, the mean content of arginine accounted for 86.91% of the total amino acids, whereas γ-aminobutyric acid accounted for 4.43%, glutamine for 2.82% and proline for 0.88%. The concentration of total nucleosides and nucleobases in YN group was significantly lower than in the other groups, where as that of essential amino acids such as lysine, tryptophan, valine in YN or YS group was lower than in GN or GS group. The differences in chemical composition across groups may be explained by differences in soil microenvironments (Supplementary Table S1).

### Variations in metabolites of *A. sinensis* under different soil microenvironments

**Fig. 2:**
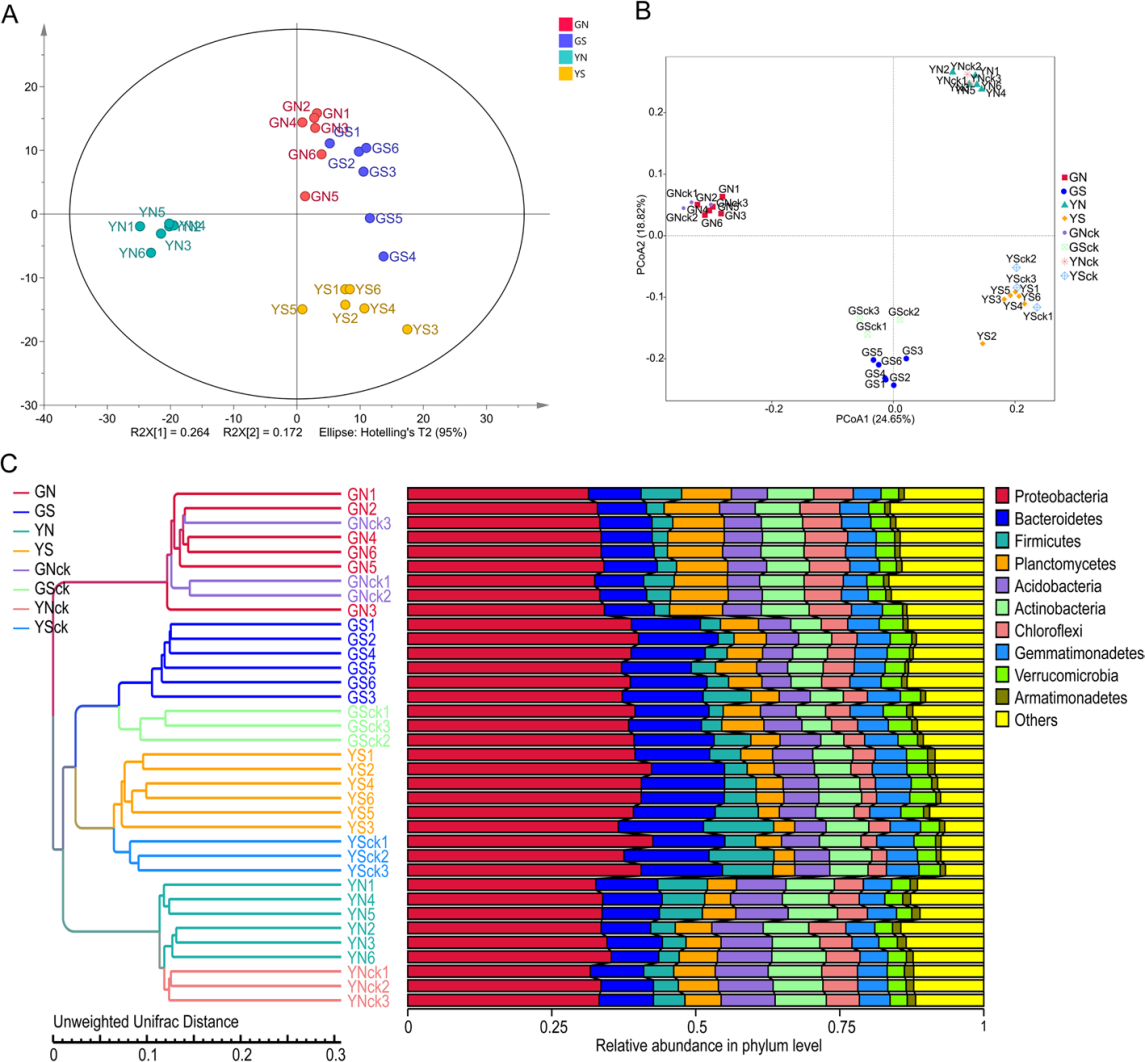
The structure of metabolites and microbial communities of *A. sinensis* samples. PCA scores for the comparison of metabolomic profiles between GN, GS, YN, YS group (**A**); principal coordinates analysis (PCoA) based on unweighted UniFrac (UUF) distance metric (**B**); and unweighted pair-group method with arithmetic mean (UPGMA) clustering analyses at phylum level of samples from GN, GS, YN, YS, GNck, GSck, YNck, YSck group (*n* = 6) (**C**)

**Fig. 3:**
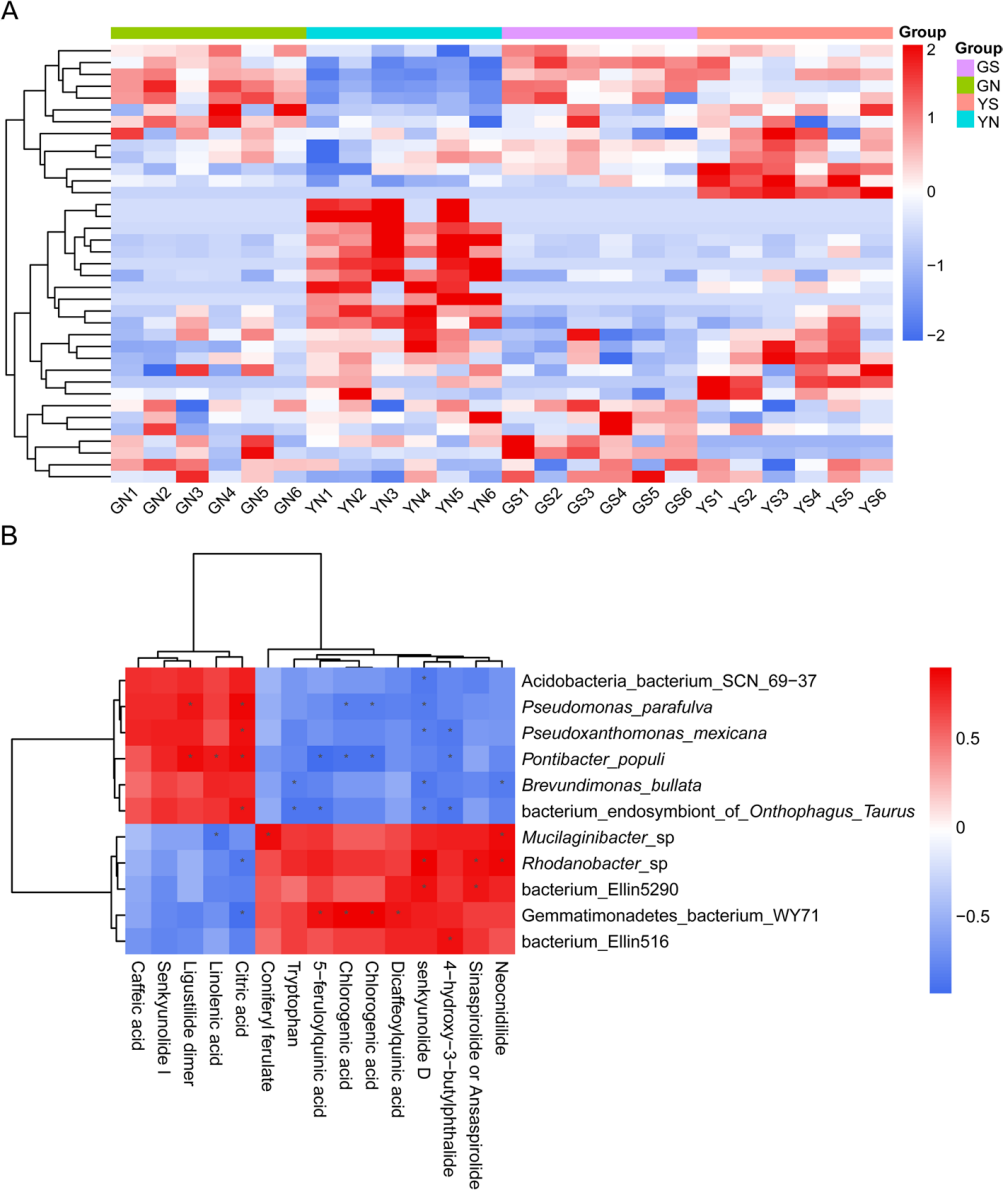
Potentially mechanistic associations between rhizosphere microbes and metabolites. Hierarchical clustering heatmap shows a consistent clustering pattern within individual groups and a diverse clustering pattern between different groups (**A**); covariation between microbes and small molecules in *A. sinensis*, specifically those of differentially abundant microbes and metabolites matched against standards between GS and YS group (Spearman’s rank correlation with two-tailed nominal *p* values) (*n *= 6) (**B**)

The quality of medicinal plants is influenced by the diversity and biochemistry of different compounds [18]. In this study, a simple, rapid and sensitive UPLC-UV-QTOF-MS/MS method was developed to characterize metabolites in *A. sinensis* samples grown under different soil microenvironments. Principal components analysis (PCA) showed that the metabolic profiles varied significantly (*p* < 0.05) across groups, and the samples were clustered into four groups: GN, YN, GS and YS groups. Notably, GS and YS samples were clustered close to GN samples, especially GS samples. However, GN, GS and YS samples formed distant clusters from YN samples (Fig. 2A). The R^2^X, R^2^Y and Q^2^Y of the OPLS-DA model were 0.667, 0.966 and 0.851, respectively. The results of permutation analysis confirmed the stability and reliability of the OPLS-DA model. The hierarchical clustering heatmap showed that the molecular features within groups had similar clustering patterns, whereas molecular features between groups showed differences, which was consistent with the PCA clustering pattern (Fig. 3A).

In the UPLC-QTOF-MS/MS analysis, both positive and negative ion modes were utilized for qualitative identification. A total of 44 metabolites were identified by chemical standards or tentatively identified by comparing their quasi-molecular ions, empirical molecular formulas, and/or fragment ions with those of known chemicals (Supplementary Table S2) [19]. We selected markers that contributed greatly to the grouping by setting the Variable Importance for Projection (VIP) value greater than 1.5 and the *p*-value less than 0.05 in the Moderated t-Test. In general, the contents of ligustilide dimers was higher in plants of GN and GS groups, whereas the content of chlorogenic acid showed an opposite trend. More importantly, the plants of GN group had higher levels of ligustilide dimers, but lower levels of unknown [19], senkyunolide A and chlorogenic acid compared to YN group. Further analysis revealed that plants of GS group had higher levels of ligustilide dimers, *Z*-6,7-epoxyligustilide and senkyunolide I, while the level of chlorogenic acid and dicaffeoylquinic acid was lower compared to YS group. The results demonstrated that the plants in non-sterilized groups had higher levels of ligustilide dimers than those of the sterilized groups (Supplementary Fig. S3 and S2, Supplementary Table S3).

### Annotation and community composition of rhizosphere bacteria under different soil microenvironments

The V4 region of 16S rRNA gene in the total 36 samples was amplified using PCR and sequenced using the IonS5^TM^XL platform. An average of 80,118 clean reads per sample were obtained. High-quality reads were clustered into 10,583 OTUs based on > 97% sequence identity, and then annotated using Silva132 database. A total of 65 OTUs were annotated at the phylum level, accounting for 87.60% of all OTUs; and 721 OTUs were annotated at the genus level. At the phylum level, we found that *Proteobacteria* (relative abundance of 55.38%), *Bacteroidetes* (10.65%) and *Acidobacteria* (7.26%) were the dominant species. At the genus level, the roots were dominated by *Stenotrophomonas* (9.97%), *Rhodanobacter* (4.19%) and *Lactobacillus* (1.48%).

**Fig. 4:**
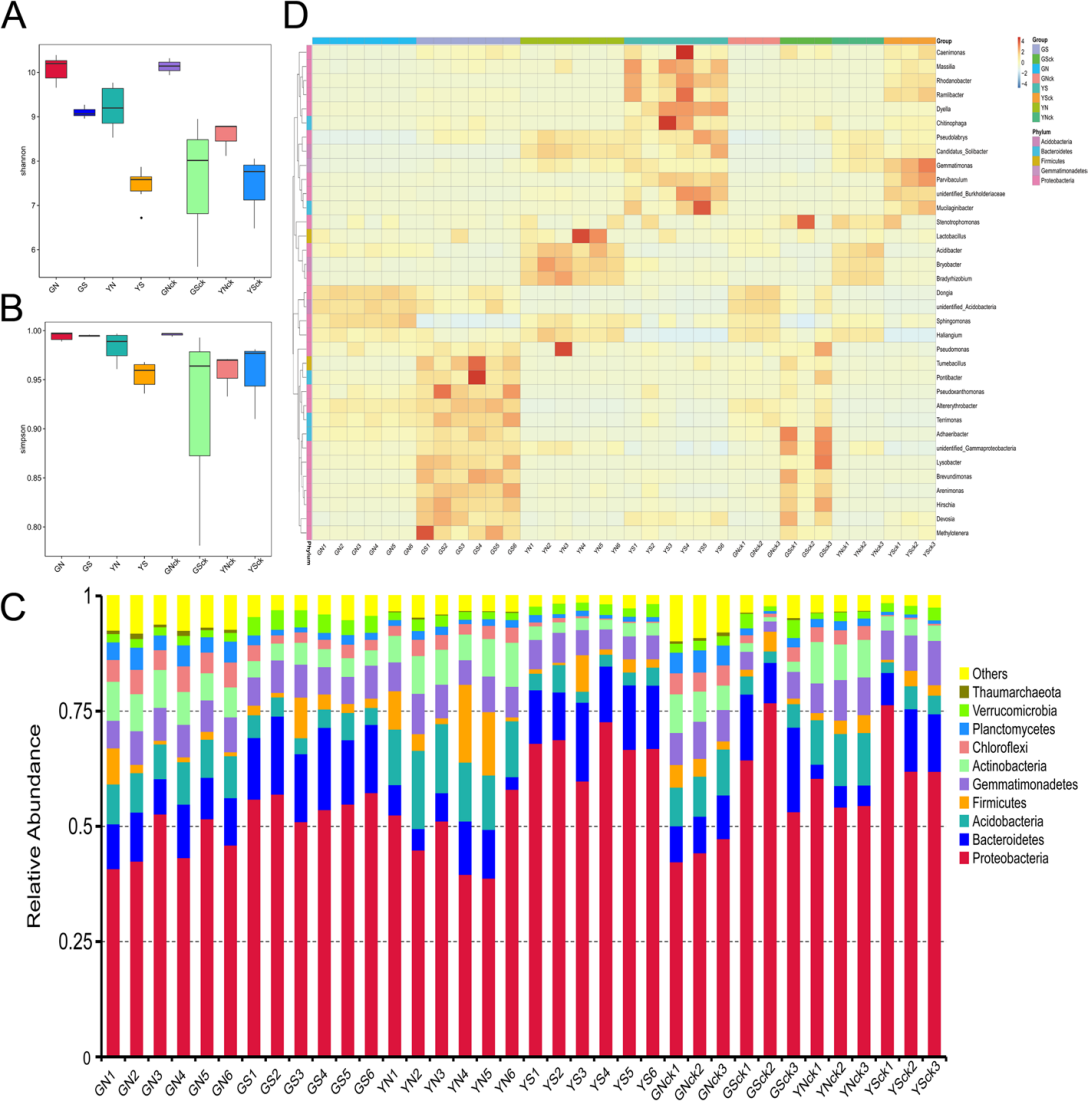
The composition of bacteria rhizosphere microbial communities. Shannon index (**A**); simpson index (**B**); Phylum distribution (**C**); heatmap distribution and hierarchical clustering at the genus level (**D**) of samples from GN, GS, YN, YS, GNck, GSck, YNck, YSck group (*n* = 6)

α-diversity refers to the microbial diversity of a sample, similar conclusions were drawn by calculating the Chao1, Shannon's diversity index and Simpson diversity index, etc. (Fig. 4A and B). The results revealed higher community diversity in the non-sterilized groups than in the sterilized groups, and higher community diversity in the Gansu groups than in Yunnan groups. However, community diversity was not different between the planting groups and the corresponding non-planting groups. Results of ANOSIM showed that the difference between groups was significantly (*p* < 0.05) greater than that within groups, indicating that the results were reliable. Collectively, these results indicated that the diversity of the bacterial communities of *A. sinensis* was influenced by the soil microenvironments, and the plant effect had little influence.

PCoA based on weighted UniFrac (WUF) distance metric showed that the community composition of the planting groups and corresponding non-planting groups were highly similar. The sterilized and non-sterilized samples separated along the first axis, and PC1 explained 38.65% of the total variation, indicating that indigenous bacteria were the most important factor affecting the rhizosphere bacterial community. Samples from Gansu and Yunnan were clearly distinguished on the second axis, and PC2 explained 23.35% of the total variation, indicating that geographical location were the second most important factor contributing to variations in rhizosphere bacterial community. However, when using an unweighted UniFrac (UUF) distance, geographic location showed largest contribution to the variation in the tested factors, and indigenous bacteria were the second most important source of variation (Fig. 2B).

The results of the PCoA were consistent with findings from unweighted pair-group method with arithmetic mean (UPGMA) clustering (Fig. 2C). Hierarchical clustering based on UUF distance metric showed complete clustering of samples in GN, GS, YN, and YS groups. However, it was observed that planting groups and the corresponding non-planting groups clustered together, indicating that plants had limited influence on the overall community composition. Samples from GS and YS initially clustered into one branch, then to YN samples, and finally the GN samples. This indicated that the GN group was different from the other three groups. To provide statistical support the above analysis, ADONIS and MRPP were employed to perform statistical tests on samples from different groups, and similar conclusion was reached.

### Comparison of composition of rhizosphere bacteria across groups

To fully understand the distribution of OTU among groups, we constructed Venn diagrams, and used them to calculate the proportion of uniquely identified OTUs in each group and OTUs shared between different groups. It was found that the proportion of OTUs shared between GN and GNck groups, YN and YNck groups, GN and YN groups, GS and YS groups, GN and GS groups, YN and YS groups accounted for 48.74%, 42.17%, 38.17%, 30.10%, 35.88%, and 32.50% of all OTUs, respectively. This showed high similarity in terms of microbial community structure between the planting groups and corresponding non-planting groups. A Venn diagram for all planting groups, showed that the number of unique OTUs belonging to the GN group was the highest, with 1692 species; and that of the YS group was the least, with 226 species. The same phenomena were recorded in all non-planting groups.

At the phylum level, the top 12 most abundant phylum from each sample or group were used to generate a accumulative column diagram showing the relative abundance of all species. The results showed that the microbial community was dominated by *Proteobacteria*, *Bacteroidetes,* and *Acidobacteria*. Moreover, a significantly (*p* < 0.05) higher relative abundance of *Proteobacteria* and *Bacteroidetes*, and a significantly (*p* < 0.05) lower relative abundance of *Acidobacteria*, *Actinobacteria*, and *Chloroflexi* was found in all sterilized groups compared to non-sterilized groups. The relative abundance of *Chloroflexi* and *Planctomycetes* was significantly (*p* < 0.05) higher in YN group, while that of *Acidobacteria* was significant (*p* < 0.05) lower than in GN group. In addition, the relative abundance of *Gemmatimonadetes*, *Actinobacteria*, and *Chloroflexi* was higher*,* while the relative abundance of *Proteobacteria* was lower in the GS group than in the YS group. The differences in microbial community between planting groups and corresponding non-planting groups was not significant (*p* > 0.05). Figure 4C shows the distribution of phylum level.

At the genus level, 721 taxa were identified from all 36 samples. The roots of *A. sinensis* were preferentially colonized by *Stenotrophomonas*, *Rhodanobacter* and *Lactobacillus*. A heatmap constructed based on hierarchical clustering of top 35 most abundant genera showed (Fig. 4D) that there were distinct differences in microbial community structure and relative abundance of most dominant genera across groups. Of note, the relative abundance of *Lysobacter* and *Brevundimonas* in Gansu group was significantly (*p* < 0.05) higher, whereas that of *Rhodanobacter* and *Gemmatimonas* was significantly (*p* < 0.05) lower than in Yunnan group. In addition, the relative abundance of *Sphingomonas* was higher in the GN group than in YN group, and the opposite was true for *Mucilaginibacter*. The relative abundance of *Pseudomonas* was higher in GS group than in YS group; while that of *Ramlibacter* and *Sphingomonas* showed opposite results. Of note, the relative abundance of *Sphingomonas* in all non-sterilized groups was significantly (*p* < 0.05) higher than in the corresponding sterilized groups.

### Putative mechanisms regulating the relationship between microbes and quality-linked metabolites

The secondary metabolites of *A. sinensis* are mainly composed of phthalides, organic acids, and polysaccharides, among which phthalides are the main bioactive ingredients, with diverse structural features [2, 20]. Previously, we found that *Pseudomonas*, *Lysobacter*, *Pseudoxanthomonas*, and *Brevundimonas* were significantly (*p* < 0.05) enriched in GS group than in the YS group. The contents of ligustilide dimer and senkyunolide I in GS group were significantly (*p* < 0.05) higher than in YS group, and were strongly positively correlated with *Pseudomonas parafulva*, *Pseudoxanthomonas maxicana*, and *Brevundimonas bullata* (average Spearman r > 0.5) (Fig. 3B). Among them, ligustilide dimer was extremely significantly (*p* < 0.05) positively correlated with *P. parafulva* (Spearman r = 0.82) (Supplementary Table S4). We therefore speculated that the high abundance of *P. parafulva* in the GS group contributed to the high levels of phthalides and was beneficial to the growth and quality improvement of *A. sinensis*.

Some ferulic acid derivatives such as ferulic acid ester have stronger biological activity and lower toxicity. In this study, organic acids and their esters were significantly (*p* < 0.05) enriched in YS group. This indicated that chlorogenic acid, dicaffeoylquinic acid and 5-feruloylquinic acid were positively associated with *Gemmatimonadetes bacterium* WY71 (average Spearman r > 0.8); coniferyl ferulate was positively associated with *Mucilaginibacter* sp. (Spearman *r* = 0.85) (Fig. 3B) (Supplementary Table S4) [21]. *Mucilaginibacter* has been found to be abundant in the rhizosphere soil of *Angelica* medicinal plants. For example, *M. herbaticus* sp nov. [22], *M. polysacchareus* sp nov. [23], and *M. angelicae* sp nov [24]. were isolated from the rhizosphere, root surface of *A. sinensis*, and rhizosphere of *A. polymorpha* Maxim, respectively. We speculated that the high abundance of *Mucilaginibacter* in YS soil might be due to the high level of ferulic acid derivatives.

## Discussion

### Driving factors that determine the quality of *A. sinensis* under different soil conditions

Considerable reports indicate that the quality of Chinese drugs is significantly associated with geographical differences potentially attributed to the variation of altitude, climate, soil properties, and rhizospheric microorganisms, etc. Herein, we revealed that the soil microenvironment is a significant factor contributing to the quality improvement of *A. sinensis*. The tradition holds that higher the contents of ferulic acid and *Z*-ligustilide is associated with better quality of the radix *A. Sinensis.* Compared to the YN and GS groups, the GN group displayed significantly (*p* < 0.05) larger dry weight, root diameter, and root length (Table 1), whereas the contents of chlorogenic acid and *Z*-ligustilide in Yunnan groups were significantly (*p* < 0.05) higher than that of Gansu groups (Table 2). Moreover, the content of senkyunolide I, butylidenephthalide, ligustilide dimers were higher in Gansu groups compared to the Yunnan groups, whereas ferulic acid demonstrated a decreasing trend (Table 1, Supplementary Table S3). Zhang et al. found that the content of ferulic acid and levistolide A in non-geoherbs regions was higher than that of geoherbs regions, whereas senkyunolide I and butylidenephthalide demonstrated an opposite trend [19], which concur with the present conclusions. Contrarily, a large-scale field study found that the root length and head diameter showed a negative correlation with ferulic acid, and there was a negative association between root length and *Z*-ligustilide [25]. However, the evaluation of the quality of radix *A.sinensis* based on their appearance traits was inaccurate.

Phenolic acids have widely been reported as key allelochemicals of medicinal plants, including coumalic acid, ferulic acid, vanillic acid, 4-hydroxybenzoic acid, and syringic acid, which are found in root exudates of *P. ginseng*, *R. glutinosa*, and *Pinellia ternata* [26]. Imperatorin, vanillin, and ferulic acid are potential allelopathic autotoxic substances in rhizosphere soil and were found to significantly inhibit the growth of *A. sinensis* seedlings [27]. The high content of ferulic acid in plants of Yunnan groups indicates that these allelochemicals largely accumulate in the Yunnan soils, consequently deteriorating the growth of *A. sinensis*. Similarly, higher content of ferulic acid has been reported in sterilized groups than in non-sterilized groups, which may justify the weak growth of plants in the sterilized groups.

### Effect of soil type on the composition of rhizosphere bacteria of *A. sinensis*

A continental-scale study on soil bacterial populations revealed that the richness and diversity of soil bacterial communities differ by ecosystem type, in particular, soil pH was the largest source of variation [28]. Compared to loessial soil and continuous cropping soil, polysaccharides and glucose amounts were significantly (*p* < 0.05) higher in *Dioscorea opposita* Thunb. growing in sandy soil. Moreover, the low soil pH was potentially associated with a high level of galacturonic acid from continuous cropping soil [29]. The soil of Gansu was slightly alkaline, whereas that of Yunnan was weakly acidic. Notably, variation in soil pH was attributed to significant differences in the rhizosphere and root-associated plant microbiomes. In the GN group, the relative abundance of *Sphingomonas*, *Lysobacter,* and *Pseudoxanthomonas* were higher than that of the YN group, whereas that of *Rhodanobacter*, *Gemmatimonas,* and *Mucilaginibacter* decreased significantly (*p* < 0.05) (Fig. 4D). Compared to the YS group, the relative abundance of *Pseudomonas*, *Lysobacter* and *Pseudoxanthomonas* were higher in the GS group, whereas that of *Rhodanobacter*, *Ramlibacter*, *Dyella* showed an opposite trend (Fig. 4D). Wu et al. found that the relative abundance of *Lysobacter*, *Pseudoxanthomonas*, *Pseudomonas,* and *Burkholderia* were significantly (*p* < 0.05) higher in the newly cultivated soil of *R. glutinosa* compared to continuous monocropping soil [30]. Similarly, the abundance of beneficial bacteria including *Arthrobacter*, *Burkholderia*, *Rhodanobacter*, and *Sphingobacterium* was negatively correlated with the accumulation of toxic DiBP in monocropping soil of *P. ginseng* [12]. Therefore, we deduced that the genus *Lysobacter*, *Pseudomonas,* and *Burkholderia,* etc. are potentially essential microbes for radix or rhizoma medicinal herbs.

Previously, *Sphingomonas* [31], *Lysobacter* [32, 33], and *Pseudoxanthomonas* [34, 35] were found to promote resistance to diseases and insect pests, as well as the degradation of harmful substances in the soil. Additionally, *Sphingomonas* promoted plant absorption through carbohydrate secretion into the rhizosphere [31]. *Lysobacter* strains exerted strong antagonistic activity against several pathogens, including *Rhizoctonia solani*, *Fusarium oxysporum,* and *Xanthomonas carnpestris*, etc.) [32]. Additional studies revealed that the genus *Pseudomonas* belongs to a group of plant growth-promoting rhizobacteria (PGPR) [36, 37]. For instance, *Pseudomonas* acted as a key PGPR in the rhizosphere of garlic regardless of growth periods, soil type, and agricultural practices. The constructed six *Pseudomonas* strains displayed strong plant growth-promoting effects [38]. These findings implied that stronger growth characteristics of *A. sinensis* in the Gansu group may be related to the high rhizosphere abundance of these antagonistic microorganisms.

### Effects of indigenous microorganisms on rhizosphere bacterial communities and growth of *A. sinensis*

Many years of planting sole crops, including grain crops, vegetable crops, medicinal plants, etc., are frequently associated with widespread continuous cropping challenges. Speculation had it that soil sterilization could influence the physical structure and microbiological properties of soil. Subsequently, this triggered the nutrient release and inhibition of soil pathogens, thereby promoting plant growth. In the present study, *A. sinensis* from sterilized replant soil demonstrated higher plant performance and enhanced activities of active oxygen scavenging enzymes. Such effects may be driven by the increased number and diversity of culturable microbial populations and functional bacterial groups [39]. While soil sterilization could relieve the soil succession cropping failure, it eliminated indigenous microbes, including a few pathogens, and limited the potential beneficial effects of soil microbes to host plants [40]. Some reports show that indigenous microorganisms are critical in plant growth and agricultural productivity, and have potential benefits in plant secondary metabolism [41]. For instance, inoculation with PGPR *Bacillus flexus* alleviated damage from host salinity stress through modification of its physiological and biochemical state, for example, promoting photosynthesis, osmotic regulation, antioxidant enzymatic activities, and regulation of Na^+^/K^+^ homeostasis [42].

Herein, we found that plants grown in sterilized soil displayed worse growth and a low survival rate, which could be attributed to the newly cropped soil (Table 1). This validated the prediction that indigenous microorganisms are essential players in plant growth and development. Likewise, *Glycyrrhiza uralensis* grown in sterilized soil displayed poorer plant growth and photosynthesis. Inoculation with AM (Arbuscular mycorrhizae) fungi in sterilized soil compensated for the absence of indigenous microbial communities, which consequently promoted plant growth and increased glycyrrhizin and liquiritin contents [40]. Moreover, we found that the relative abundance of *Sphingomonas* was significantly (*p* < 0.05) higher in the GN group compared to the GS group, whereas *Ramlibacter*, *Gemmatimonas,* and *Brevundimonas* demonstrated an opposite trend (Fig. 4D). The relative abundance of *Sphingomonas* and *Bradyrhizobium* in the YN group was higher than that of the YS group, whereas *Rhodanobacter*, *Ramlibacter,* and *Dyella* were significantly (*p* < 0.05) lower (Fig. 4D). Additionally, *Bradyrhizobium* was revealed as a PGPR and could secrete plant hormones and QS metabolites, regulating the symbiotic relationship with the host and the adaptation to environmental variations [43]. QS-deficient (unable to produce cinnamoyl-HSL) mutant *Bradyrhizobium* sp. strains exhibited significantly (*p* < 0.05) lower colonization potential in rice roots and reduced plant growth compared to the wild-type strain [44]. The high abundance of beneficial bacteria, including *Sphingomonas* and *Bradyrhizobium*, in the rhizosphere of non-sterilized groups, accounted for better growth and quality of *A. sinensis* in the unsterilized groups.

### Potentially mechanistic association of rhizosphere microbes with metabolites

Many plant endophytes can synthesize various secondary metabolites, some of them show good biological activities, which help to quality improvement of medicinal plants [45]. Inoculation with endophytes isolated from the alkaloid-rich genotype of *Catharanthus roseus* remarkably improves ajmalicine and serpentine contents in roots of low alkaloid yielding genotype [46]. The small molecules, including amino acids, organic acids, sugars, and secondary metabolites secreted by roots can promote the growth of some soil microorganisms, initiating the migration of soil microbial communities [47]. For instance, *Falciphora oryzae* colonized Arabidopsis roots through sensory signaling molecules derived from the roots. Subsequently, it could promote the lateral root growth of *Arabidopsis* through the biosynthesis of indole derivatives [48]. Therefore, we speculated that rhizosphere microbes may have a direct or indirect association with the growth and metabolism process of the host. Particularly, PGPR from *Platycodon grandiflorum* [49], mycorrhizal fungi from *P. ginseng* [50], and Actinomycetes from *Curcuma longa* [51] significantly promoted plant growth and resistance. Furthermore, the metabolite profiling of *A. sinensis* and microbial community composition in the rhizosphere soil exhibited similar variation patterns across groups. These observations implied that there may exist a specific correlation between the secondary metabolites of *A. sinensis* and its rhizosphere microorganisms.

Furthermore, our study found that higher contents of ligustilide dimers and senkyunolide I in the GS group demonstrated a positive correlation with *P. parafulva*, *P. maxicana*, and *B. bullata* (average Spearman r > 0.5). Higher contents of chlorogenic acid, dicaffeoylquinic acid, 5-feruloylquinic acid in the YS group were positively correlated with *Gemmatimonadetes bacterium* WY71 (average Spearman r > 0.8). Similarly, positive association between coniferyl ferulate and *Mucilaginibacter* sp. (Spearman r = 0.85) (Supplementary Table S4). Of note, this positive association between a metabolite and species may denote that the metabolite promotes the growth of the species in question, or that the species produces that metabolite [52]. Studies have revealed that particular microorganisms can induce the accumulation of metabolites by (i) elevating the expression of genes associated with the synthesis of secondary metabolites, (ii) activating the host's defense responses, and (iii) synthesis of crucial enzymes that convert precursors into effective constituents [53]. A study found that *Pseudomonas* sp. and *Pantoea* sp. in the rhizosphere could stimulate the plant growth and synthesize of major phenolic acids in *Salvia miltiorrhiza* through its production of the abundant types and contents of phytohormones [54]. Elsewhere, inoculation of *Trichoderma asperellum* significantly increased the artemisinin concentration and dry weight of *Artemisia annua* L. leaves through elevated expression of artemisinin biosynthesis crucial enzymatic genes, *HMGR1*, *FPS*, *ADS*, *CYP71AV1*, *CPR*, *DBR*, *DXS1,* and *DXR1* [55]. Dark Septate Endophytes (DSE) could establish a symbiotic association with liquorice, increasing its plant N and P absorption, biomass, and accumulation of glycyrrhizic acid and glycyrrhizin [56]. The *P. parafulva* genome encod a series of antibacterial secondary metabolites, including lipopeptides, pyridine, benazine, and hydrogen cyanide, and exerts excellent antagonistic effects against rice and soybeans plant pathogens including *Rhizoctonia solani, Xanthomonas axonopodis,* and *Burkholderia glumae* [57, 58]. Further studies should explore whether the observed population structure stabilizes over time or is correlated with changes in metabolomic or microbiome composition in *A. sinensis.* Also, scholars should identify the plant genes associated with these taxa and extensively characterize their functions [7].

## Conclusion

This study explored changes in metabolite profiling and rhizosphere microbial communities of *A. sinensis* under different soil microenvironments. It further provides a few insights into the mechanism by which rhizosphere microorganisms impact the quality improvement of authentic *A. sinensis*. The present findings affirm that the survival rate and growth status of *A. sinensis* in Gansu soils are better than that of Yunnan soils, higher in unsterilized groups than sterilized groups. High abundance of beneficial bacteria, including *Sphingomonas*, *Pseudomonas*, *Lysobacter*, *Pseudoxanthomonas* in Gansu soils and the enhanced growth characteristics of *A. sinensis* from Gansu soils demonstrated that the rhizosphere microbiome is an essential plant requirement for normal growth and development. Furthermore, ligustilide dimers enriched in the GS group demonstrated a notable positive correlation with the relative abundance of *P. parafulva.* Organic acids enriched in the YS group were positively correlated with the relative abundance of *Gemmatimonadetes bacterium* WY71 and *Mucilaginibater* sp. Collectively, these findings offer a foundation for further exploration of the relationship between rhizosphere microbes and the growth and quality formation of *A. sinensis,* and its mechanism. Such an approach is key in elucidating the mechanism of the association of microorganisms with the growth of medicinal plants.

## Materials and Methods

### Pot experiment site and design

Soils for testing were sampled from the experimental base of the Min Conty Medicinal Plants Growing Technology Extension Centre (34°22′30" N, 104°53′20" E; black soil, pH ≈ 8.0) and the Medicinal Plants Research Institute Yunnan Academy of Agricultural Sciences (26°28 ′42" N, 100°4′34" E; red soil, pH ≈ 6.5), whereby *Vicia faba* L. was the previously planted crop. The soil samples were transported to Shili Town of Min County, Gansu Province, with an average annual rainfall of 635.0 ± 17.4 mm, an annual average temperature of 5.43 ± 0.71 °C, and annual mean sunshine duration of 2154.3 ± 68.7 h. After sieving (2 mm), the soil samples were divided into two: One portion was sterilized by autoclaving at 121 °C for 4 h, whereas the other portion was not sterilized. The *A. sinensis* seedlings (Gansu danggui 90–01) provided by Min County Xizhai town Zhucai herbs Planting Professional Cooperative, were transplanted during the local traditional planting period. We set the experiment into eight groups, including four experimental groups (GN, GS, YN, YS) and four control groups (GN-ck, GS-ck, YN-ck, YS-ck). The soil without seedling implant acted as a control group ( six repeats of each group).

The soil was put into a large plastic box and mixed thoroughly with a base fertilizer (diamine phosphate 10.6 g per pot, organic fertilizer 23.6 g per pot). The mixed soil was put to 4 ~ 6 cm lower the container mouth and compacted. Then, 8 seedlings were planted in each pot, with a depth of 3 cm and an interval of about 4 cm. The seeds were covered with topsoil and a small amount of water (about 50 mL) was applied to moisten the seeding layer. During the whole trial period, no fungicides were sprayed, manual weeding and pest control were timely completed. When the soil layer 2 cm below the topsoil dried, we irrigated each pot with the same amount of water. In 2018, *A. sinensis* plants were left to grow for 180 days. Carefully, the fresh plants were uprooted and gently shaken to remove loosely attached soil [30]. The rhizosphere soil tightly attached to tuberous roots was collected. Fresh plants and soil samples were transferred to the laboratory in an ice cooler and stored at -80 °C awaiting analyses.

### DNA extraction, PCR amplification, and sequencing

In total, 36 soil samples collected from the 8 groups of the pot experiment (GN, GS, YN, YS, GN-ck, GS-ck, YN-ck, YS-ck) were shipped to 100,015 (China) Beijing Novogene Biotech Co., Ltd. for 16S rRNA gene amplicon sequencing using the Ion S5™ XL platform. We employed the CTAB or SDS method to extract genomic DNA. To check for DNA purity and concentration, agarose gel electrophoresis was used. Then DNA was diluted with sterile water to 1 ng·μL^−1^. The variable region V4 of the bacterial 16S rRNA gene was amplified using degenerate PCR primers 515F and 806R (Walters et al. 2016). The samples were separated on a 2% (w/v) agarose gel, and mixed in equivalent amounts based on the PCR product concentration. The mixed PCR products were purified using the GeneJET™ Gel Extraction Kit (Thermo Scientific). Briefly, 16S rRNA gene amplicons were extracted using Ion Plus Fragment Library Kit 48 rxns (Thermo Scientific) following the manufacturer's instructions. We sequenced the built DNA amplicon library on the Ion S5™ XL platform after Qubit quantification and examination of genomic libraries.

### Data processing and analysis

The raw sequencing data from Ion AmpliSeq were processed using Cutadapt (V 1.9.1) (Martin M., 2011). The barcode and primer sequence for preliminary quality control were eliminated to obtain raw reads. To get clean reads, the chimera sequences were detected and deleted from the dataset using the UCHIME algorithm. The clean reads were clustered into operational taxonomic units (OTUs) at a sequence similarity level of 97% using Uparse software (v7.0.1001). Taxonomic annotation via the SSUrRNA database was performed using Mothur (version 1.25.1) and SILVA132 (http://www.arb-silva.de/). The MUSCLE (V 3.8.31) software was employed to perform multiple sequence alignment to establish the phylogenetic relationship of all OTUs sequences. The QIIME (V 1.9.1) and R software (V 2.15.3) were applied for sample diversity analysis. α-diversity was evaluated considering the OTU tables. Spearman rank correlation analysis was conducted in R using the vegan packages.

### A multi-index comprehensive evaluation of the chemical composition of *A. sinensis*

#### Method 1: Preparation of sample solutions for secondary metabolite analysis

Exactly 0.2 g dried powder (40 mesh) was sonicated (100 Hz, 25 °C) with 4 mL 70% methanol for 45 min in a 50 mL glass-stoppered conical flask. The samples were centrifuged (12,000 r/min, 10 min). The supernatants were injected directly onto an A Thermo Syncronis C_18_ (2.1 mm × 100 mm, 1.7 μm) column. The mobile phase constituted A (0.1% formic acid) and B (acetonitrile). The optimized elution conditions included: 0 ~ 2 min, 5% ~ 10% B; 2 ~ 10 min, 10% ~ 40% B; 10 ~ 14 min, 40% ~ 58% B; 14 ~ 20 min, 58% ~ 90% B; 20 ~ 22 min, 90% ~ 90% B; 22 ~ 22.5 min, 90% ~ 5% B; 22.5 ~ 24 min, 5% ~ 5% B. The flow rate was 0.4 mL·min^−1^, column temperature was kept at 35 °C, with was 2 μL injection volume. Mass spectrometric detection was performed on a Waters ACQUITY™ Synapt Q-TOF mass spectrometer equipped with an electrospray ionization (ESI) source. ESI–MS spectra were generated in both positive (ESI^+^) and negative (ESI^−^) ion modes through scanning from 100 to 1000 Da. The optimized conditions are referenced to published articles in Chemistry Central Journal [59].

#### Method 2: Preparation of nucleosides, nucleobases, and amino acids analysis samples and standard solutions for qualitative and quantitative analyses

Referenced to published articles in Molecules [17]. A mixed standard stock solution of 28 reference compounds was prepared in methanol/water (9:1, v/v), with concentrations as follows: 2'-deoxyguanosine 3.6667 μg·mL^−1^, cytidine 4.3750 μg·mL^−1^, 2'- deoxycytidine 4.9167 μg·mL^−1^, cytidine-5'-monophosphate 4.1250 μg·mL^−1^, 2'-deoxyadenosine 3.9167 μg·mL^−1^, adenosine 4.3750 μg·mL^−1^, guanosine 4.0833 μg·mL^−1^, inosine 3.5417 μg·mL^−1^, uridine 5.2083 μg·mL^−1^, ornithine 3.6333 μg·mL^−1^, leucine 15.0000 μg·mL^−1^, iso-leucine 15.0000 μg·mL^−1^, phenylalamine 15.3333 μg·mL^−1^, tryptophan 15.8333 μg·mL^−1^, aminobutyric acid 19.0000 μg·mL^−1^, methionine 17.0000 μg·mL^−1^, proline 18.5000 μg·mL^−1^, valine 17.1667 μg·mL^−1^, trans-4-hydroxy-L-proline 21.0000 μg·mL^−1^, glutamincacid 15.6667 μg·mL^−1^, glutamine 13.8333 μg·mL^−1^, asparagine 23.8333 μg·mL^−1^, tyrosine 15.3333 μg·mL^−1^, threonine 17.3333 μg·mL^−1^, lysine 18.5000 μg·mL^−1^, serine 15.5000 μg·mL^−1^, citrulline 17.3333 μg·mL^−1^, and arginine 32.3333 μg·mL^−1^. The working standard solution for the calibration curves were prepared by diluting the mixed standard solution with 10% methanol at different concentrations.

#### Method 3: Preparation of organic acids and phthalides analysis samples and mixed control solutions

Here, 0.2 g dried powder (40 mesh) was extracted in the ultrasound-assisted condition at 25 °C with 20 mL methanol for 40 min in a 50 mL glass-stoppered conical flask. After centrifugation (13,000 r/min, 10 min) and filtration (0.22 μm membrane filter), the supernatants were injected into the UPLC system for analysis. A mixed standard stock solution of 9 reference compounds was prepared in methanol/water (9:1, v/v), with concentrations as follows: chlorogenic acid 24.1313 mg·L^−1^, ferulic acid 15.8838 mg·L^−1^, senkyunolide I 7.5600 mg·L^−1^, senkyunolide H 3.0208 mg·L^−1^, coniferyl ferulate 18.0340 mg·L^−1^, senkyunolide A 5.3025 mg·L^−1^, n-butylphthalide 199.9788 mg·L^−1^, Z-ligustilide 17.9196 mg·L^−1^, butylidenephthalide 15.0 mg·L^−1^. The working standard solution for calibration curves was prepared by diluting the mixed standard solution with methanol at different concentrations. The separation was done on a Thermo Syncronis C_18_ (2.1 mm × 100 mm, 1.7 μm) column. The mobile phase constituted: A (0.1% formic acid) and B (acetonitrile) with a gradient elution: 0 ~ 2 min, 5% ~ 10% B; 2 ~ 10 min, 10% ~ 40% B; 10 ~ 13 min, 40% ~ 40% B; 13 ~ 19 min, 40% ~ 50.8% B; 19 ~ 20 min, 50.8% ~ 90% B; 20 ~ 21 min, 90% ~ 90% B; 21 ~ 23 min, 90% ~ 5% B. Column temperature was maintained at 35 °C; the flow rate was 0.4 mL·min^−1^, with a detection wavelength of 260 nm, 280 nm, 320 nm and injection volume of 2 μL. Linear regression models between sample concentration and peak area were established to generate linear regression equation and linear ranges of chlorogenic acid, ferulic acid, senkyunolide I, senkyunolide H, coniferyl ferulate, senkyunolide A, n-butylphthalide, Z-ligustilide, and butylidenephthalide respectively.

### Statistical analysis

All statistical analyses were performed in R software (V 2.15.3) (R Foundation for Statistical Computing, Vienna, Austria) and IBM SPSS 20.0 (IBM Corporation, USA). Analysis of variance (ANOVA) and multiple comparison analysis was applied to calculate the mean and standard deviation and for statistical tests. The differences of parameter variance were estimated by ANOVA and the Kruskal Wallis rank-sum test based on the distribution of parameter statistics. For post-hoc comparison, Tukey’s honest significant differences tests and Wilcoxon matched-pairs signed-rank test were applied. R (V 3.2.2) was employed to perform heat map, PCoA, and hierarchical clustering. The R language did a comparison of microorganisms through the ‘adonis’ function from R’s ‘vegan’ package. The multiple null hypothesis testing of the prior group was achieved using the ‘anosim’ or ‘adonis’ function from R’s ‘vegan’ package. According to the method of Franzosa et al. [52], we clustered the differentially abundant features via a custom approach. Then, the correlation analysis of the differentially abundant chemical components with the differentially abundant microorganisms was undertaken (For all clustering analyses, we applied Spearman’s rank correlation as a similarity measure with a threshold of r = 0.7). Unless otherwise stated, the significance level was set at *p* < 0.05.

## Supplementary Information

**Additional file 1: Table S1.** Comparison of the nucleosides, nucleobases, and essential amino acids contents in different groups of *A. sinensis* samples.

**Additional file 2: Table S2.** Secondary metabolites identified in *A. sinensis* by untargeted metabolomics.

**Additional file 3: Table S3.** Tentative markers for discriminating *A. sinensis* samples between every two groups. Note: A, GN and YN group; B, GS and YS group; C, GN and GS group; D, YN and YS group. **Figure S1.** OPLS-DA scores (A), permutation Test of OPLS-DA model (B), S-Plot (C), and VIP value (D) for the comparison of metabolomic profiles between GN and YN group in positive ion mode. **Figure S2.** OPLS-DA scores (A), permutation Test of OPLS-DA model (B), S-Plot (C), and VIP value (D) for the comparison of metabolomic profiles between GS and YS group in positive ion mode.

**Additional file 4: Table S4.** Spearman’s rank correlation scores of representative metabolites and representative species between GS and YS group.

## Data Availability

The datasets used and/or analysed during the current study are available from the corresponding author on reasonable request.

## Abbreviations

TCM: Traditional Chinese medicine
DiBP: Diisobutyl phthalate
QS: Quorum sensing
QQ: Quorum quenching
UPLC-UV-QTOF-MS/MS: Ultra Performance Liquid Chromatography—Ultra Violet—Quadrupole Time of Flight—Mass/Mass
OPLS-DA: Orthogonal partial least-squares discrimination analysis
PCA: Principal components analysis
VIP: Variable importance for projection
OTU: Operational taxonomic units
ANOSIM: Analysis of similarities
PCoA: Principal coordinates analysis
WUF: Weighted UniFrac
UUF: Unweighted UniFrac
UPGMA: Unweighted pair-group method with arithmetic mean
ADONIS: Permutational multivariate analysis of variance
MRPP: Multi Response Permutation Procedure
PGPR: Plant growth-promoting rhizobacteria
AM: Arbuscular mycorrhizae
DSE: Dark Septate Endophytes
ANOVA: Analysis of varia
