# Supplementary material for: Insights into the mechanism of the effects of rhizosphere microorganisms on the quality of authentic Angelica sinensis under different soil microenvironments
Source: BMC Plant Biol. 2021 Jun 22;21:285. doi: 10.1186/s12870-021-03047-w (PMC8220839; doi:10.1186/s12870-021-03047-w)
Supplement: Supplementary file 1 — Additional file 1: Table S1. Comparison of the nucleosides, nucleobases, and essential amino acids contents in different groups of A. sinensis samples. [file 12870_2021_3047_MOESM1_ESM.docx]

Table S1(A) Comparison of the nucleosides and nucleobases contents in different groups of *A. sinensis* samples (µg/g).

| Compounds | YN | YS | GN | GS |
| --- | --- | --- | --- | --- |
| Guanine | 0.09 | 0.11 | 0.05 | 0.05 |
| Cytidine-5'-monophosphate | 12.58 | 19.74 | 10.14 | 10.01 |
| 2'-Deoxyadenosine | 0.69 | 1.87 | 0.05 | 0.26 |
| 2'-Deoxycytidine | 0.14 | 0.41 | 0.08 | 0.12 |
| 2'-Deoxyguanosine | 0.45 | 0.76 | 0.28 | 0.33 |
| Adenosine | 4.17 | 6.52 | 19.04 | 17.28 |
| Cytidine | 1.58 | 1.58 | 2.80 | 2.65 |
| Guanosine | 1.97 | 4.19 | 7.56 | 7.39 |
| Inosine | 0.26 | 0.87 | 1.75 | 1.69 |
| Uridine | 4.25 | 7.26 | 9.43 | 9.32 |
| Total nucleosides and nucleobases | 26.17 | 43.30 | 51.16 | 49.09 |

Table S1(B) Comparison of the essential amino acids contents in different groups of *A. sinensis* samples (µg/g).

| Compounds | YN | YS | GN | GS |
| --- | --- | --- | --- | --- |
| Lysine | 4.72 | 4.88 | 7.14 | 5.80 |
| iso-Leucine | 1.17 | 1.35 | 1.44 | 1.72 |
| Leucine | 1.86 | 1.92 | 1.64 | 1.80 |
| Methionine | 1.82 | 1.67 | 1.68 | 1.70 |
| Phenylalamine | 1.58 | 1.65 | 1.88 | 2.02 |
| Threonine | 2.17 | 3.32 | 3.09 | 2.79 |
| Tryptophan | 2.27 | 5.22 | 6.08 | 4.94 |
| Valine | 2.76 | 2.72 | 3.68 | 3.93 |
| Total essential amino acids | 18.36 | 22.72 | 26.63 | 24.70 |
