# Supplementary material for: Insights into the mechanism of the effects of rhizosphere microorganisms on the quality of authentic Angelica sinensis under different soil microenvironments
Source: BMC Plant Biol. 2021 Jun 22;21:285. doi: 10.1186/s12870-021-03047-w (PMC8220839; doi:10.1186/s12870-021-03047-w)
Supplement: Supplementary file 2 — Additional file 2: Table S2. Secondary metabolites identified in A. sinensis by untargeted metabolomics. [file 12870_2021_3047_MOESM2_ESM.docx]

Table S2. Secondary metabolites identified in *A. sinensis* by untargeted metabolomics.

| Identification | t_R_(min) | Theoretical  accurate mass (m/z) | Q-TOF-MS (m/z)  (ESI^+^/ESI^-^) | Mass  accuracy  (ppm) | MS/MS fragment ion (m/z) |
| --- | --- | --- | --- | --- | --- |
| Citric acid | 1.10 | 191.0192[M-H]^-^ | 191.0179 [M-H]^-^ | -6.81 | - |
| Adenine | 1.52 | 136.0623[M+H]^+^ | 136.0615[M+H]^+^ | -5.88 | - |
| Adenosine | 1.52 | 268.1046[M+H]^+^ | 268.1030[M+H]^+^ | -5.97 | 136.0623[M+H-C_5_H_8_O_4_]^+^ |
| L(+)-Arginine | 2.25 | 175.1195[M+H]^+^ | 175.1180[M+H]^+^ | -8.57 | - |
| Tryptophan | 3.07 | 205.0977[M+H]^+^ | 205.0966[M+H]^+^ | -5.36 | 188.0689 [M+H-NH_3_]^+^, 146.0593[M+H-COOCH_3_]^+^ |
| Caffeic acid | 3.22 | 179.0344[M-H]^-^ | 179.0355[M-H]^-^ | 6.14 | 135.0452[M-H-CO_2_]^-^ |
| Ferulic acid 4-O-glucoside | 3.72 | 355.1029[M-H]^–^ | 355.1017[M-H]^–^ | -3.38 | 193.0490[M-H-C_6_H_10_O_5_]^-^, 178.0296, 134.0394 |
| Chlorogenic acid | 4.00 | 353.0873[M-H]^-^ | 353.0858[M-H]^-^ | -4.25 | 191.0543[M-H-C_9_H_6_O_3_]^-^ |
| \| Vanillic acid \| \| --- \| \| | 4.43 | 167.0344[M-H]^-^ | 167.0337[M-H]^-^ | -4.19 | 123.0443[M-H-CO_2_]^-^ |
| 5-Feruloylquinic acid | 5.20 | 367.1029[M-H]^-^ | 367.1022[M-H]^-^ | -1.91 | 191.0561[M-H-C_10_H_8_O_3_]^-^, 173.0439 |
| Ferulic qcid | 6.20 | 193.0501[M-H]^-^ | 193.0490[M-H]^-^ | -5.70 | 178.0266[M-H-CH_3_]^–^, 149.0626[M-H-CO_2_]^–^ |
| E-6,7-dihydroxydihydroligustilide | 6.22 | 225.1127[M+H]^+^ | 225.1118[M+H]^+^ | -4.00 | 566.4275([M+2Glu-H_2_O]^+^), 588.4107([M+2Glu-H_2_O+Na]^+^) |
| Dicaffeoylquinic acid | 6.76 | 515.1190[M-H]^-^ | 515.1180[M-H]^-^ | -1.94 | 353.0874[M-H-C_9_H_6_O_3_]^-^, 191.0546 |
| Dicaffeoylquinic acid | 7.02 | 515.1190[M-H]^-^ | 515.1183[M-H]^-^ | -1.36 | 353.0872[M-H-C_9_H_6_O_3_]^-^, 191.0543 |
| Senkyunolide I | 7.59 | 207.1021[M+H-H_2_O]^+^ | 207.1005[M+H-H_2_O]^+^ | -7.73 | - |
| Senkyunolide H | 7.95 | 207.1021[M+H-H_2_O]^+^ | 207.1008[M+H-H_2_O]^+^ | -6.28 | 191.1059 [M+2H-2H_2_O]^+^ |
| (1S)-2-O-Z-feruloyl-1-(4-hydroxyphenyl)ethane-1,2-diol | 8.87 | 329.1025[M-H]- | 329.1013[M-H]- | -3.65 | 193.0472[M-H-C_8_H_8_O_2_]^-^, 135.0442[M-H-C_10_H_10_O_4_]^-^ |
| Senkyunolide D | 9.65 | 221.0814[M-H]^-^ | 221.0808[M-H]^-^ | -2.71 | - |
| Senkyunolide B or C | 9.84 | 205.0865[M+H]^+^ | 205.0848[M+H]^+^ | -8.29 | 187.0818[M+H-H_2_O]^+^ |
| 4-Hydroxy-3-butylphthalide | 9.86 | 207.1021[M+H]^+^ | 207.1009[M+H]^+^ | -5.79 | - |
| Senkyunolide F | 10.06 | 207.1021[M+H]^+^ | 207.1013[M+H]^+^ | -3.86 | - |
| Valerophenone-o-carboxylic acid | 11.69 | 205.0865 [M-H]^-^ | 205.0855[M-H]^-^ | -4.88 | 179, 1610[M-H-CO_2_]^-^ |
| p-Hydroxyphenethyl trans-ferulate | 11.79 | 313.1076 [M-H]^-^ | 313.1066[M-H]^-^ | -3.19 | 193.0495[M-H-C_8_H_8_O]^-^, 149.0620, 134.0366 |
| Z-6,7-epoxyligustilide | 12.28 | 207.1021[M+H]^+^ | 207.1005[M+H]^+^ | -7.73 | 189.0931[M+H-H_2_O]^+^, 171.0816, 145.1024 |
| Coniferyl ferulate | 12.83 | 379.1158 [M+Na]^+^ | 379.1155 [M+Na]^+^ | -0.79 | 163.0747[M+H-C_10_H_10_O_4_]^+^ |
| Senkyunolide A | 13.49 | 193.1229[M+H]^+^ | 193.1219[M+H]^+^ | -5.18 | 175.1107[M+H-H_2_O]^+^, 137.0591[M+H-C_4_H_8_]^+^ |
| Butylphthalide | 13.80 | 191.1072[M+H]^+^ | 191.1061[M+H]^+^ | -5.76 | 173.0950[M+H-H_2_O]^+^, 145.1003[M+H-HCOOH]^+^, 135.0440[M+H-C_4_H_8_]^+^ |
| E-butylidenephthalide | 14.13 | 189.0916[M+H]^+^ | 189.0909[M+H]^+^ | -3.70 | 171.0802[M+H-H_2_O]^+^, 133.0282[M+H-C_4_H_8_]^+^ |
| E-Ligustilide | 14.45 | 191.1072[M+H]^+^ | 191.1062[M+H]^+^ | -5.23 | 173.0950[M+H-H_2_O]^+^, 145.1003[M+H-HCOOH]^+^ |
| Neocnidilide | 14.82 | 195.1385[M+H]^+^ | 195.1372[M+H]^+^ | -6.66 | 177.1266[M+H-H_2_O]^+^, 149.1320[M+H-HCOOH]^+^ |
| Z-Ligustilide | 14.99 | 191.1072[M+H]^+^ | 191.1062[M+H]^+^ | -5.23 | 173.0950[M+H-H_2_O]^+^, 145.1003[M+H-HCOOH]^+^ |
| Z-butylidenephthalide | 15.13 | 189.0916[M+H]^+^ | 189.0900[M+H]^+^ | -8.46 | 171.0791[M+H-H_2_O]^+^, 143.0851[M +H-H_2_O-CO]^+^ |
| Unknown | 15.25 | - | 520.3395[M+H]^+^ |  | 502.3278, 478.2918, 337.2714, 184.0714 |
| Unknown | 15.58 | - | 520.3400[M+H]^+^ |  | 502.3275, 483.2603, 337.2720, 184.0726 |
| Ligustilide dimer | 17.18 | 381.2066[M +H]^+^ | 381.2052[M +H]^+^ | -3.67 | 363.1981[M+H-H_2_O]^+^, 191.1052[M+H-C_12_H_14_O_2_]^+^ |
| Sinaspirolide or Ansaspirolide | 17.63 | 379.1909[M +H]^+^ | 379.1888[M +H]^+^ | -5.54 | 333.1872[M+H-HCOOH]^+^, 191.1061[M+H-C_12_H_12_O_2_]^+^ |
| Ligustilide dimer | 18.52 | 381.2066[M +H]^+^ | 381.2045[M+H]^+^ | -5.51 | 363.1941[M+H-H_2_O]^+^, 191.1060[M+H-C_12_H_14_O_2_]^+^ |
| Ligustilide dimer | 18.66 | 381.2066[M +H]^+^ | 381.2043[M +H]^+^ | -6.03 | 363.1942[M+H-H_2_O]^+^, 335.1981[M+H-HCOOH]^+^, 191.1066[M+H-C_12_H_14_O_2_]^+^ |
| Ligustilide dimer | 18.71 | 381.2066[M +H]^+^ | 381.2062[M +H]^+^ | -1.05 | 363.1956[M+H-H_2_O]^+^, 191.1063[M+H-C_12_H_14_O_2_]^+^ |
| Ligustilide dimer | 19.42 | 381.2066[M +H]^+^ | 381.2054[M +H]^+^ | -3.15 | 191.1054[M+H-C_12_H_14_O_2_]^+^ |
| Senkyunolide P | 19.53 | 383.2222[M +H]^+^ | 383.2222[M +H]^+^ | 0.00 | 338.2240[M+H-COOH]^+^, 191.1058[M+H-C_12_H_16_O_2_]^+^ |
| Ligustilide dimer | 20.05 | 381.2066[M +H]^+^ | 381.2056[M +H]^+^ | -2.62 | 191.1054[M+H-C_12_H_14_O_2_]^+^ |
| Linolenic acid | 20.79 | 277.2168[M-H]^-^ | 277.2166[M-H]^-^ | -0.72 | - |
| Linoleic acid | 22.26 | 279.2324 [M-H]^-^ | 279.2316[M-H]^-^ | -2.86 | - |
