# Supplementary material for: Insights into the mechanism of the effects of rhizosphere microorganisms on the quality of authentic Angelica sinensis under different soil microenvironments
Source: BMC Plant Biol. 2021 Jun 22;21:285. doi: 10.1186/s12870-021-03047-w (PMC8220839; doi:10.1186/s12870-021-03047-w)
Supplement: Supplementary file 3 — Additional file 3: Table S3. Tentative markers for discriminating A. sinensis samples between every two groups. Note: A, GN and YN group; B, GS and YS group; C, GN and GS group; D, YN and YS group. Figure S1. OPLS-DA scores (A), permutation Test of OPLS-DA model (B), S-Plot (C), and VIP value (D) for the comparison of metabolomic profiles between GN and YN group in positive ion mode. Figure S2. OPLS-DA scores (A), permutation Test of OPLS-DA model (B), S-Plot (C), and VIP value (D) for the comparison of metabolomic profiles between GS and YS group in positive ion mode. [file 12870_2021_3047_MOESM3_ESM.docx]

Table S3(A). Tentative markers for discriminating *A. sinensis* samples between GN and YN group.

| VIP | t_R_(min) | Mean measured mass (Da) | Theoretical exact Mass (Da) | Mass  accuracy  (ppm) | Identification |
| --- | --- | --- | --- | --- | --- |
| 7.80 | 15.25 | 520.3390 | - | - | Unknown |
| 5.01 | 13.49 | 193.1219 | 193.1229 | -5.18 | Senkyunolide A |
| 3.88 | 15.58 | 520.3391 | - | - | Unknown |
| 2.84 | 18.66 | 381.2043 | 381.2066 | -6.03 | Ligustilide dimer |
| 2.57 | 13.80 | 191.1072 | 191.1061 | -5.76 | Butylphthalide |
| 2.42 | 4.00 | 355.1029 | 355.0989 | -11.26 | Chlorogenic acid |
| 2.23 | 20.05 | 381.2056 | 381.2066 | -2.62 | Ligustilide dimer |
| 1.99 | 8.87 | 329.1013 | 329.1025 | -3.65 | (1S)-2-O-Z-feruloyl-1-(4-hydroxyphenyl)ethane-1,2-diol |

Table S3(B). Tentative markers for discriminating *A. sinensis* samples between GS and YS group.

| VIP | t_R_(min) | Mean measured mass (Da) | Theoretical exact Mass (Da) | Mass  accuracy  (ppm) | Identification |
| --- | --- | --- | --- | --- | --- |
| 4.25541 | 18.71 | 381.2068 | 381.2066 | 0.52 | Ligustilide dimer |
| 4.22779 | 3.07 | 205.0966 | 205.0977 | -5.26 | Tryptophan |
| 3.20549 | 18.66 | 381.2043 | 381.2066 | -6.03 | Ligustilide dimer |
| 3.17932 | 4.00 | 355.0993 | 355.1029 | -10.14 | Chlorogenic acid |
| 3.08919 | 12.28 | 207.1005 | 207.1021 | -7.73 | Z-6,7-epoxyligustilide |
| 2.78915 | 14.44 | 191.1062 | 191.1072 | -5.23 | E-Ligustilide |
| 2.24389 | 20.05 | 381.2056 | 381.2066 | -2.62 | Ligustilide dimer |
| 2.23268 | 17.18 | 381.2052 | 381.2066 | -3.67 | Ligustilide dimer |
| 2.20007 | 7.59 | 207.1005 | 207.1021 | -7.73 | Senkyunolide I |

Table S3(C). Tentative markers for discriminating *A. sinensis* samples between GN and GS group.

| VIP | t_R_(min) | Mean measured mass (Da) | Theoretical exact Mass (Da) | Mass  accuracy  (ppm) | Identification |
| --- | --- | --- | --- | --- | --- |
| 7.56 | 15.58 | 520.3391 | - | - | Unknown |
| 4.68 | 18.71 | 381.2068 | 381.2066 | 0.52 | Ligustilide dimer |
| 4.53 | 18.66 | 381.2043 | 381.2066 | -6.03 | Ligustilide dimer |
| 2.97 | 14.44 | 191.1062 | 191.1072 | -5.23 | E-Ligustilide |
| 2.25 | 20.06 | 381.2056 | 381.2066 | **-**2.62 | Ligustilide dimer |
| 2.15 | 17.18 | 381.2052 | 381.2066 | -3.67 | Ligustilide dimer |
| 1.98 | 2.41 | 175.1180 | 175.1195 | -8.57 | L(+)-Arginine |

Table S3(D). Tentative markers for discriminating *A. sinensis* samples between YN and YS group.

| VIP | t_R_(min) | Mean measured mass (Da) | Theoretical exact Mass (Da) | Mass  accuracy  (ppm) | Identification |
| --- | --- | --- | --- | --- | --- |
| 6.38 | 14.99 | 191.1062 | 191.1072 | -5.23 | Z-Ligustilide |
| 6.36 | 15.25 | 520.3390 | - | - | Unknown |
| 4.67 | 13.49 | 193.1219 | 193.1229 | -5.18 | Senkyunolide A |
| 3.98 | 15.58 | 520.3391 | - | - | Unknown |
| 3.55 | 18.71 | 381.2068 | 381.2066 | 0.52 | Ligustilide dimer |
| 3.20 | 18.66 | 381.2043 | 381.2066 | -6.03 | Ligustilide dimer |
| 3.06 | 3.07 | 205.0966 | 205.0977 | -5.26 | Tryptophan |
| 2.57 | 13.80 | 191.1061 | 191.1072 | -5.76 | Butylphthalide |
| 2.13 | 20.05 | 381.2056 | 381.2066 | -2.62 | Ligustilide dimer |
| 2.05 | 2.26 | 175.1180 | 175.1195 | -8.57 | L(+)-Arginine |


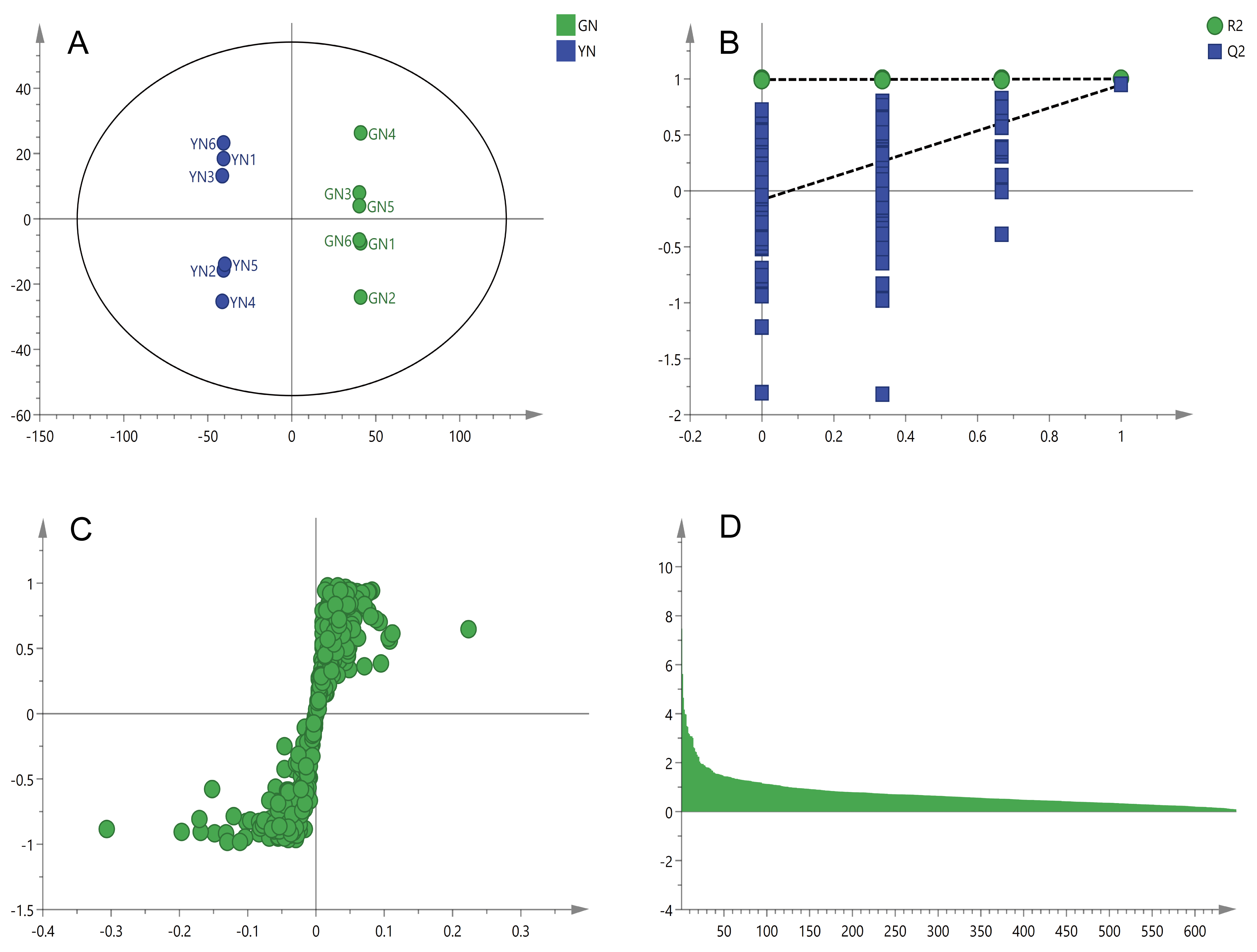


Figure S1


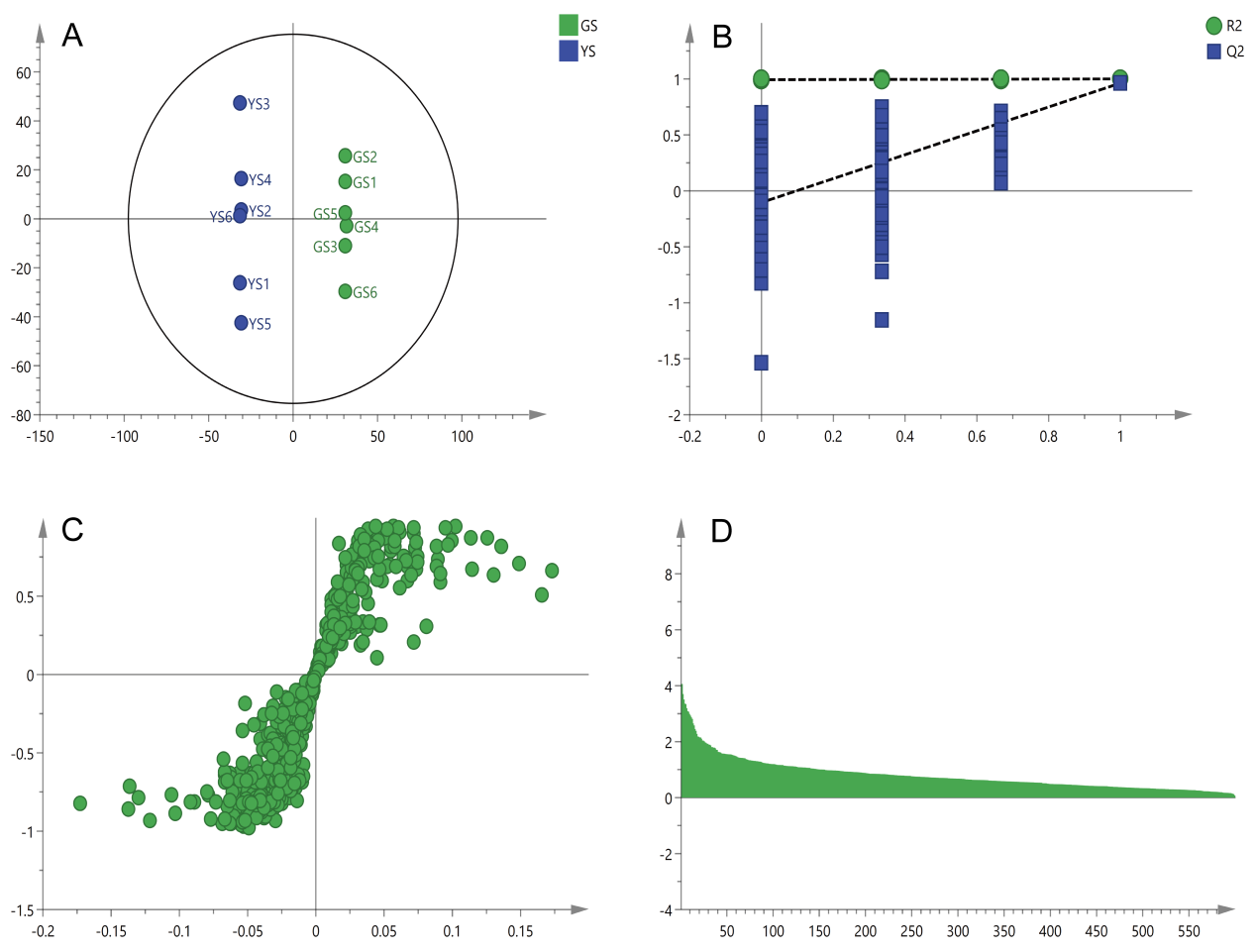


Figure S2
