# Supplementary material for: Insights into the mechanism of the effects of rhizosphere microorganisms on the quality of authentic Angelica sinensis under different soil microenvironments
Source: BMC Plant Biol. 2021 Jun 22;21:285. doi: 10.1186/s12870-021-03047-w (PMC8220839; doi:10.1186/s12870-021-03047-w)
Supplement: Supplementary file 4 — Additional file 4: Table S4. Spearman’s rank correlation scores of representative metabolites and representative species between GS and YS group. [file 12870_2021_3047_MOESM4_ESM.docx]

Table S4. Spearman’s rank correlation scores of representative metabolites and representative species between GS and YS group.

|  | Chlorogenic acid | Caffeic acid | Senkyunolide I | Tryptophan | Dicaffeoylquinic acid | Linolenic acid | Citric acid | Sinaspirolide or Ansaspirolide | Neocnidilide | 5-feruloyl  quinic acid | Senkyunolide D | Coniferyl ferulate | 4-hydroxy-3- butylphthalide | Ligustilide dimer |
| --- | --- | --- | --- | --- | --- | --- | --- | --- | --- | --- | --- | --- | --- | --- |
| *Rhodanobacter* sp | 0.6993 | -0.4965 | -0.6706 | 0.7343 | 0.6713 | -0.7483 | -0.8462 | 0.8266 | 0.8741 | 0.7762 | 0.8920 | 0.6084 | 0.7353 | -0.4965 |
| *Pseudomonas parafulva* | -0.8231 | 0.7426 | 0.7499 | -0.6655 | -0.7951 | 0.6690 | 0.8652 | -0.6991 | -0.6795 | -0.7250 | -0.8338 | -0.5219 | -0.7590 | 0.8231 |
| *Pontibacter populi* | -0.8807 | 0.5825 | 0.7669 | -0.7614 | -0.7123 | 0.8000 | 0.8561 | -0.5752 | -0.7614 | -0.9333 | -0.7865 | -0.6632 | -0.8315 | 0.8491 |
| *Brevundimonas bullata* | -0.6434 | 0.5035 | 0.6472 | -0.8112 | -0.5315 | 0.7622 | 0.7343 | -0.7642 | -0.8392 | -0.7832 | -0.8472 | -0.5874 | -0.7950 | 0.6014 |
| *Mucilaginibacter* sp | 0.5544 | -0.4246 | -0.5947 | 0.6842 | 0.6246 | -0.8737 | -0.7123 | 0.7825 | 0.8597 | 0.6982 | 0.7678 | 0.8456 | 0.7753 | -0.5965 |
| *Acidobacteria* bacterium SCN 69-37 | -0.6713 | 0.7203 | 0.7018 | -0.6643 | -0.7343 | 0.6434 | 0.7832 | -0.7876 | -0.6923 | -0.5944 | -0.8547 | -0.4895 | -0.7577 | 0.7483 |
| *Pseudoxanthomonas mexicana* | -0.7684 | 0.7544 | 0.7708 | -0.7860 | -0.7053 | 0.5860 | 0.8246 | -0.6534 | -0.6632 | -0.6947 | -0.8128 | -0.4807 | -0.8540 | 0.7684 |
| *Gemmatimonadetes* bacterium WY71 | 0.8963 | -0.5800 | -0.7643 | 0.6643 | 0.8084 | -0.6995 | -0.9034 | 0.6742 | 0.6749 | 0.8331 | 0.7729 | 0.5975 | 0.7617 | -0.7874 |
| Bacterium Ellin5290 | 0.5394 | -0.5534 | -0.7343 | 0.4904 | 0.7811 | -0.7811 | -0.7881 | 0.8124 | 0.7601 | 0.6480 | 0.8412 | 0.5814 | 0.6992 | -0.5079 |
| Bacterium Ellin516 | 0.6620 | -0.6900 | -0.7773 | 0.6620 | 0.7531 | -0.5814 | -0.7916 | 0.6952 | 0.6025 | 0.5990 | 0.7665 | 0.5009 | 0.8263 | -0.7461 |
| Bacterium endosymbiont of O nthophagus Taurus | -0.7549 | 0.5891 | 0.7159 | -0.8678 | -0.6032 | 0.7055 | 0.8113 | -0.6215 | -0.7796 | -0.8642 | -0.8133 | -0.5468 | -0.8736 | 0.6702 |

Only signifificant correlations (*p* < 0.05) are shown.
